# Diseases and Displacements of the the Uterus

**Published:** 1872-10

**Authors:** 


### Books Reviewed.

*Diseases and Displacements of the Uterus.* By Edwin Nesbit Chapman, M. A., M. D. New York: William Wood & Co. Buffalo: T. Butler & Son, 1872.

It appears that on being called to the Chair of Clinical Midwifery in the Long Island Medical College, twelve years since, Prof. Chapman found it necessary to institute a thorough course of clinical study, with the view to "unravel the mysteries of female diseases," and that this work is based mainly upon original clinical observations then made, with a reproduction of his clinical lectures on the diseases and displacements of the uterus.

A careful perusal of his reported cases is instructive. He makes a very broad distinction between inflammation and congestion, claiming that congestion of uterus, ovaries and vagina is the frequent cause of nearly all the symptoms of uterine disease. Leeching, Scarification and Caustic are his favorite remedies. When the congestion is relieved, all symptoms disappear.

The conditions of disease and displacement are very plainly described, and a mode of treatment detailed, which, in his hands, proved very successful. We think that every physician having care of similar cases, will be deeply interested in his clinical commentaries upon cases, as well as in his general descriptions of uterine disease. His idea of studying uterine diseases afresh, strikes us with special favor. He has contributed valuable clinical cases, and his treatment appears satisfactory in most instances, but we are not yet willing to believe but valuable knowledge may still be gained by studying uterine diseases and uterine therapeutics anew.

